# TBK1 is part of a galectin 8 dependent membrane damage recognition complex and drives autophagy upon Adenovirus endosomal escape

**DOI:** 10.1371/journal.ppat.1010736

**Published:** 2022-07-20

**Authors:** Noémie Pied, Coralie F. Daussy, Zoé Denis, Jessica Ragues, Muriel Faure, Richard Iggo, Mario P. Tschan, Benoit Roger, Fabienne Rayne, Harald Wodrich

**Affiliations:** 1 CNRS UMR 5234, Fundamental Microbiology and Pathogenicity, Université de Bordeaux, Bordeaux, France; 2 INSERM U1218, Institut Bergonié, University of Bordeaux, Bordeaux, France; 3 Division of Experimental Pathology, Institute of Pathology, University of Bern, Bern, Switzerland; Stony Brook University, UNITED STATES

## Abstract

Intracellular pathogens cause membrane distortion and damage as they enter host cells. Cells perceive these membrane alterations as danger signals and respond by activating autophagy. This response has primarily been studied during bacterial invasion, and only rarely in viral infections. Here, we investigate the cellular response to membrane damage during adenoviral entry. Adenoviruses and their vector derivatives, that are an important vaccine platform against SARS-CoV-2, enter the host cell by endocytosis followed by lysis of the endosomal membrane. We previously showed that cells mount a locally confined autophagy response at the site of endosomal membrane lysis. Here we describe the mechanism of autophagy induction: endosomal membrane damage activates the kinase TBK1 that accumulates in its phosphorylated form at the penetration site. Activation and recruitment of TBK1 require detection of membrane damage by galectin 8 but occur independently of classical autophagy receptors or functional autophagy. Instead, TBK1 itself promotes subsequent autophagy that adenoviruses need to take control of. Deletion of TBK1 reduces LC3 lipidation during adenovirus infection and restores the infectivity of an adenovirus mutant that is restricted by autophagy. By comparing adenovirus-induced membrane damage to sterile lysosomal damage, we implicate TBK1 in the response to a broader range of types of membrane damage. Our study thus highlights an important role for TBK1 in the cellular response to adenoviral endosome penetration and places TBK1 early in the pathway leading to autophagy in response to membrane damage.

## Author summary

Rapid detection of invading pathogens is crucial for cell survival. Membrane alterations in this process are detected by cells but are rarely studied in the context of viral infections. TBK1 is an important kinase driving innate immunity and autophagy in response to pathogen invasion. Here we report that TBK1 promotes autophagy in response to membrane penetration by adenoviruses. We demonstrate that TBK1 is rapidly activated and recruited to virus membrane penetration sites, and promotes autophagy through its kinase activity. We show that TBK1 recruitment depends on membrane damage recognition via galectin 8 but occurs independently of classical autophagy receptors or functional autophagy. Moreover, we demonstrate that TBK1 activation is part of a wider cellular response to endo-lysosomal damage. Our work highlights a prominent role for TBK1 in the swift cellular response to membrane damage and the downstream activation of autophagy.

## Introduction

The ability to detect and neutralize invading pathogens is an essential requirement to maintain cell integrity. Recognizing membrane distortions or membrane damage caused by invading pathogens permits cells to catch an invader at the onset of infection and to mount appropriate responses. Pathogens must develop countermeasures to blunt this early cellular response. Adenovirus (Ad), a non-enveloped DNA virus, is able to reach the cytosol as an almost intact particle thanks to its ability to escape from the endo-lysosomal compartment efficiently. It first enters the cell by receptor mediated endocytosis [1,2]. Once in the endosome, it penetrates the endosomal membrane to reach the cytosol, where it is transported to the nucleus by microtubule motor proteins [3–5]. Escape from the endosome is essential to avoid lysosomal degradation and triggering endosomal innate immune receptors [6]. The membrane lytic activity of Ad is carried by protein VI, a viral protein released from the capsid interior by an unknown trigger during endosomal passage [7,8]. Protein VI contains an amphipathic helix that applies positive membrane curvature to the endosomal membrane, leading to membrane rupture [7,9]. Using a hyperstable Ad mutant that fails to release protein VI from the capsid or mutating the amphipathic helix in protein VI results in reduced Ad infectivity, suggesting that endosomal escape is a highly coordinated and stepwise process [10,11].

Membrane damage, as induced during adenoviral endosomal escape, exposes endosomal intra-luminal glycans to proteins in the cytosol. Exposed glycans are recognized as danger signals by cytosolic lectins that cluster at the sites of membrane damage [12]. Several intracellular pathogens, including Ads, are known to trigger the recruitment of galectin 3 (Gal3) and galectin 8 (Gal8) to penetration sites, a finding that has been exploited to visualize the penetration event [13–15]. Both galectins also accumulate when membrane damage is caused by chemical or physical agents, indicating that recognition of membrane damage by galectins is a general cellular defense mechanism [16–19]. Several examples show that membrane penetration by pathogens triggers selective autophagy as part of a cell autonomous defense program [15]. Macroautophagy/autophagy (hereafter autophagy) is a conserved degradation pathway in which double-membraned vesicles called autophagosomes engulf the cargo to be degraded [20]. Covalent attachment of phosphatidyl-ethanolamine to cytosolic LC3 protein (microtubule associated protein 1A/1b light-chain 3) is a key step in autophagy leading to the incorporation of LC3 into the autophagosomal membrane [21,22]. Selective autophagy works through targeted (selective) cargo recognition by a group of autophagy receptors termed SLR for Sequestosome1/p62-like receptors, including p62/sequestosome1 (p62), optineurin, nuclear dot protein 52/Calcium-binding and coiled coil domain containing protein 2 (NDP52) and its homolog Tax1 binding protein 1 (Tax1BP1). Autophagy receptors link the cargo to the growing autophagosomal membrane by binding to conjugated LC3 via specific interaction domains [12,23]. Cargo that has been selectively sorted into autophagosomes in this way is degraded when the autophagosome fuses with lysosomes.

Tank binding kinase (TBK1) has recently been implicated in autophagy activation. It is recruited by autophagy receptors to damaged organelles such as mitochondria [24], aggregates [25], or lysosomes [26], leading to their elimination. TBK1 is also involved in the degradation of invasive bacteria by autophagy, following detection of cellular membrane penetration by galectins. *Salmonella enterica serovar* Typhimurium (*S*. *Typhimurium*) ruptures the vacuole in which it replicates, leading to the recruitment of Gal3, 8 and 9, although only Gal8 is able to restrict bacterial growth [13]. Gal8 forms a complex with NDP52, which recruits key factors for autophagosome formation. Gal8 is also responsible for local activation of TBK1 [27]. Activated TBK1 promotes the phosphorylation of autophagy receptors including NDP52 [27], p62 [25], optineurin [28] and Tax1BP1 [29]. Phosphorylation of the receptors is thought to drive sustained autophagy activation [28,29]. In the absence of TBK1, *S*. *Typhimurium* proliferation is strongly increased [27,28,30]. A similar role for TBK1 has been shown for *Listeria monocytogenes* [31], *Mycobacterium tuberculosis* [32] and *Streptococcus pyogenes* [33], suggesting that TBK1 plays an essential role in restricting bacterial infections following cellular membrane rupture.

Despite the progress in understanding bacterial invasion, remarkably little attention has been given to the cellular response to membrane penetration in the case of viral pathogens. Picornaviruses release their genomes from endosomes in a manner that activates Gal8-directed autophagy [34]. Endosome penetration by human adenovirus type C5 (HAd-C5) or their vector derivatives also triggers autophagy accompanied by the accumulation of NDP52 and p62 at the viral entry site [14]. Entering Ad particles are able to evade the autophagy response they induce by preventing or delaying autophagosome maturation and lysosome fusion [14]. This way, Ads are able to escape from the ruptured endosome before being cleared by autophagy. This is achieved through a short PPxY peptide motif in the membrane lytic protein VI, which is used by Ad to recruit the HECT (homologous to the E6-AP Carboxyl Terminus) ubiquitin ligase NEDD4.2 (neural precursor cell expressed developmentally down-regulated 4-like). An HAd-C5 mutant named “M1” harbors a mutation in the PPxY motif and does not interact with NEDD4.2, making it ~ 10 fold less infectious [35]. Interestingly, depletion of Gal8 or inhibition of autophagy fully rescues M1 infectivity showing that the mutant virus is indeed cleared by autophagy [14]. How Ad membrane penetration activates autophagy in the first place and the mechanism that renders Ad resistant to clearance by autophagy are currently not understood. Given its role in anti-bacterial autophagy, we asked whether TBK1 plays an analogous role in anti-adenoviral autophagy. We show that Ad membrane penetration results in rapid TBK1 phosphorylation and its recruitment to the penetration site. We further show that in Ad infected cells TBK1 phosphorylation relies on membrane damage sensing by Gal8 but not Gal3. Unexpectedly, neither classical autophagy receptors nor functional autophagy are required for TBK1 activation. We further show that TBK1, and more specifically its kinase activity, reinforces autophagy activation in response to Ad infection. Finally, we provide evidence that TBK1 is part of a comprehensive cellular machinery for sensing damaged membrane, by showing that it is also phosphorylated and recruited to sites of lysosome damage induced by LLOMe (L-leucyl-L-leucine methyl ester). Taken together our results decipher in detail how membrane damage caused by Ad is detected and highlight a prominent role for TBK1 as part of a cellular sensing complex for membrane damage and downstream autophagy activation.

## Results

### Penetration of the endosomal membrane by adenovirus activates TBK1

**Fig 1:**
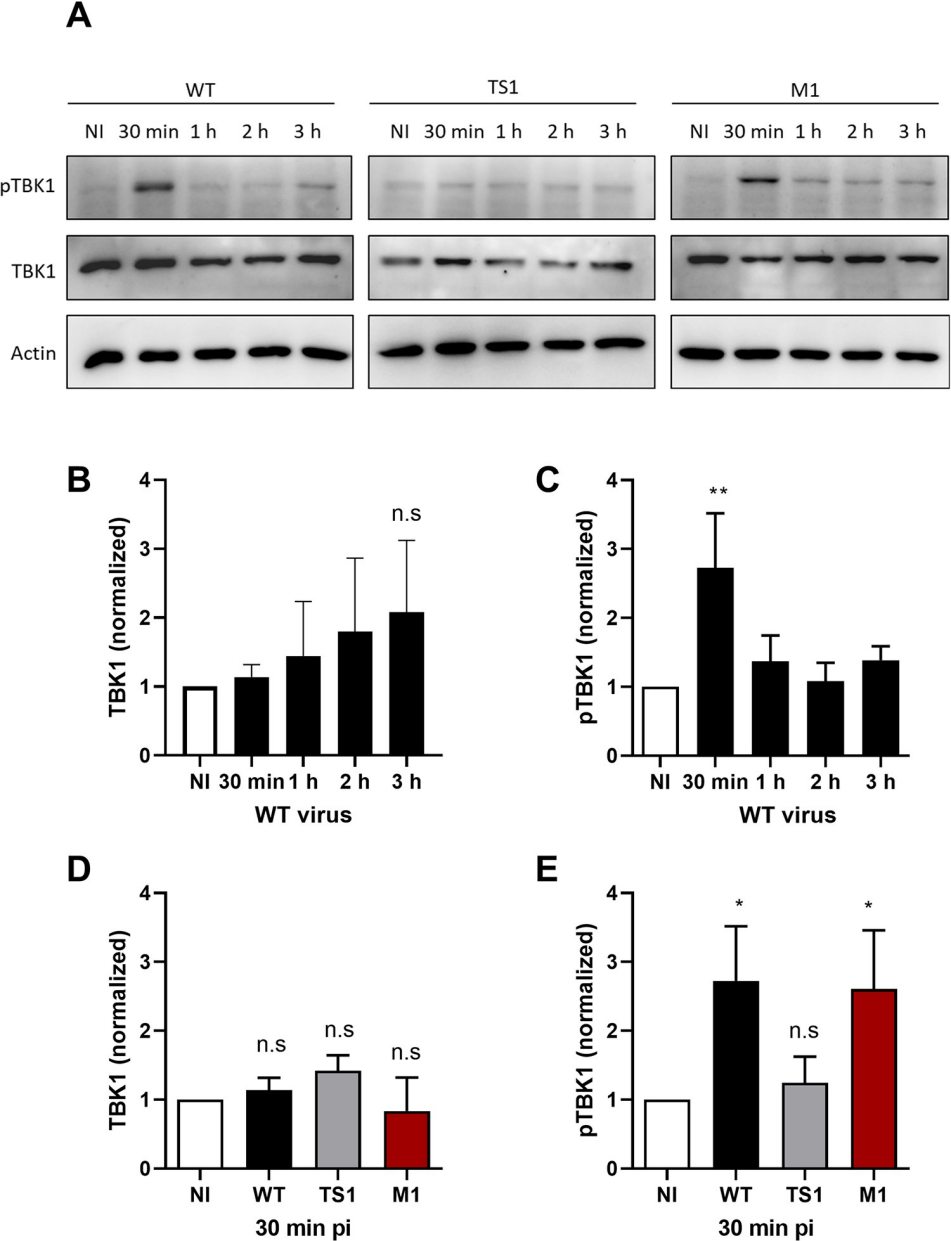
TBK1 activation is membrane damage dependent. (**A**) U2OS cells were infected with Ad WT (**left panel**), TS1 (**middle panel**) and M1 (**right panel**) and analyzed by western blot at different times pi (from 30 min to 3 h) using antibodies against S172 phosphorylated TBK1 (pTBK1), total TBK1 (TBK1) and actin as a loading control. (**B-C**) Quantification of TBK1 (**B**) or pTBK1 (**C**) signal from western blots upon Ad WT infection over time normalized to non infected (NI) conditions. (**D-E**) Quantification of TBK1 (**D**) or pTBK1 (**E**) signal from western blots upon infection with Ad WT (black), TS1 (grey) or M1 (red) at 30 min pi. Data shown are normalized to NI conditions. All data of B to E are the mean ± SD of 3 independent experiments. P values are based on Ordinary ONE-WAY ANOVA analysis and Dunnett’s multiple comparison.

We recently showed that despite many differences to bacterial invasion, HAd-C5 activates autophagy when it penetrates the endosomal membrane to reach the cytosol [14,15]. We thus started our investigation by asking if the role for TBK1 in bacterial entry is also involved in Ad entry. We first tested if TBK1 is activated upon Ad cell entry. U2OS cells were infected with a non-replicative adenoviral vector deleted for the E1/E3 regions and expressing green fluorescent protein (HAd-C5-WT-GFP, hereafter named “WT” for wild type). Infected cells were collected at different times post infection (from 30 min to 3 h pi). TBK1 activation can be followed via its phosphorylation on serine 172 (S172), referred to as pTBK1 in the text and figures [36,37]. Using western blot analysis, we were able to detect TBK1 phosphorylation as early as 30 min pi (**Fig 1A, left panel**). While total TBK1 level did not significantly change upon Ad infection (**Fig 1B**), pTBK1 levels increased ~3-fold at 30 min pi and returned to basal levels within 1 h after infection (**Fig 1C**), suggesting that Ad WT entry induces a fast and transient activation of TBK1. The TBK1 phosphorylation correlated with the peak of Ad endosomal membrane penetration [14]. To answer whether the Ad membrane penetration process is responsible for TBK1 activation, we infected cells with two Ad mutants. The first, HAd-C5-*ts1*-GFP, is a non-replicative adenoviral vector deleted for the E1/E3 regions and expressing green fluorescent protein with an additional mutation in the protease open reading frame derived from the HAd-C2-*ts1* virus. This mutation creates a maturation deficient, hyperstable virus particle, which fails to release protein VI and is not causing endosome rupture [10,11]. This mutant is named “TS1” in our study to be distinguished from the WT vector. The second, HAd-C5-M1-GFP, is a non-replicative adenoviral vector deleted for the E1/E3 regions and expressing green fluorescent protein with an additional mutation in the protein VI gene changing the PPxY motif to PGAA. This vector fails to escape from the endosomal compartment and remains associated with ruptured endosomes to be subsequently degraded by autophagy [14,35]. This mutant is named “M1” in our study to be distinguished from the WT and the TS1 vectors mentioned above. Our results show that the TS1 mutant was unable to trigger TBK1 phosphorylation (**Fig 1A, middle panel**) while the M1 mutant activated TBK1 with similar kinetics as the WT (**Fig 1A, right panel**). To compare the TBK1 activation by the different viruses, we compared levels of total TBK1 (**Fig 1D**) and pTBK1 (**Fig 1E**) at 30 min pi in three independent experiments. The analysis confirmed that total TBK1 levels remain unchanged upon infection and that TBK1 was only phosphorylated in the case of endosome penetrating WT and M1 viruses. Our analysis thus showed that TBK1 is activated during Ad entry and that viral endosome penetration is required.

### Phosphorylated TBK1 is recruited to the site of Ad endosome penetration

**Fig 2:**
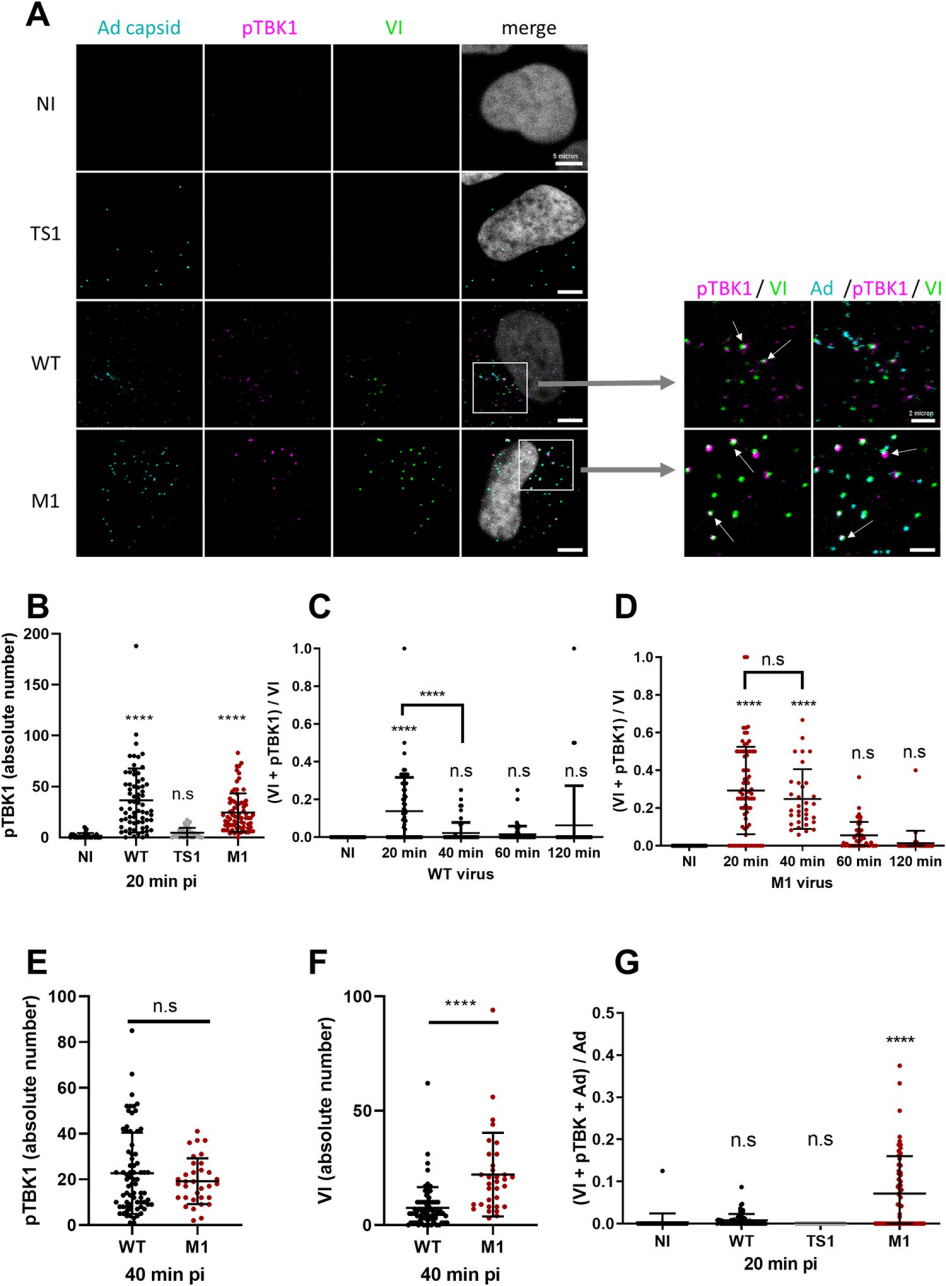
Phosphorylated TBK1 recruitment to the site of membrane damage. U2OS cells were infected with fluorescently labeled (cyan) Ad WT, TS1 or M1 and analyzed by immunofluorescence at different time pi (from 20 min to 120 min) using anti protein VI (green) and anti pTBK1 (magenta) antibodies. (**A**) Representative confocal images at 20 min pi are presented. An enlarged inset section is shown on the right indicating VI and pTBK1 double colocalizations or VI, pTBK1 and Ad triple colocalizations (arrows). (**B**) Quantification of pTBK1 foci from A (n>30). (**C**) Proportion of VI colocalization with pTBK1 over time of infection (n>30). (**D)** Same analysis as in C using M1 infected cells. (**E-F**) Quantification of pTBK1 (**E**) and VI (**F**) dots at 40 min pi for Ad WT (black), and M1 (red) (n>30). (**G**) Proportion of Ad capsid colocalization with both VI and pTBK1 for Ad WT (black), TS1 (grey) and M1(red) at 20 min pi (n>30). P values are based on Ordinary ONE-WAY ANOVA analysis and Dunnett’s multiple comparison test for B, C, D, G and unpaired t-test for E and F.

We next wanted to know if pTBK1 accumulates at the endosome penetration site or if TBK1 activation is part of a delocalized cell response to Ad entry. Adenovirus endosome penetration sites were detected with antibodies to protein VI, which is transiently exposed during the process [14,35]. We infected U2OS cells with Alexa-labeled Ad WT, TS1 and M1. At 20 min pi, cells were fixed and processed for immunofluorescence analysis using specific antibodies against protein VI (green signal) and pTBK1 (magenta signal), while virus particles (cyan signal) were directly detected (**Fig 2A, left panel**). We observed pTBK1 accumulation in cytosolic foci reminiscent of viral endosome penetration sites (**Fig 2A**). Image quantifications revealed that these foci accumulated exclusively in WT and M1 but not in TS1 infected cells or non-infected (NI) control cells (**Fig 2B**). Several pTBK1 foci colocalized with protein VI (**indicated for WT and M1 in Fig 2A right panel**) showing that pTBK1 accumulated at viral membrane penetration sites. To characterize the dynamics of pTBK1 accumulation during Ad entry, we next performed infection kinetics using WT, M1 and TS1 viruses and quantified the colocalization of pTBK1 with VI over time. Normalization of colocalization events with the total number of VI foci showed that in WT infected U2OS cells, pTBK1 was present at ~15% of Ad membrane penetration sites at 20 min pi. This association was transient and decreased over time, reaching background levels after 40 min pi (**Fig 2C**). In M1 infected cells, pTBK1 also accumulated at 20 min pi at ~30% of Ad penetration site, and remained associated with ~25% of those sites at 40 min pi (**Fig 2D**). The difference between WT and M1 was not due to differences in pTBK1 recruitment because the absolute number of pTBK1 foci per cell was similar for WT and M1 at 40 min pi (**Fig 2E**), but due to a faster decline in protein VI detection which is rapidly degraded in WT infections following endosomal escape (**Fig 2F**) [35]. We next investigated the association of pTBK1 with penetrating virus particles and looked at triple colocalizations of Ad, VI and pTBK1 to identify ruptured endosomes from which the virus has not yet escaped (**Fig 2A right panel**). Triple colocalizations were quantified 20 min pi (**Fig 2G**). Triple colocalization was absent in non-infected or TS1 infected control cells. For WT infection, very few Ad virions colocalizing with pTBK1 were present in (VI positive) ruptured endosomes reminiscent of rapid endosomal escape (**Fig 2F and 2G**). M1 virions in contrast remained associated with VI and accumulated pTBK1, explaining the prolonged association. Taken together, these results show that active TBK1 accumulates rapidly at the Ad penetration site. The association with Ad particles is transient in the case of fast escaping Ad WT and prolonged with escape defective Ad M1.

### TBK1 activation and recruitment to the site of Ad membrane penetration requires Gal8

**Fig 3:**
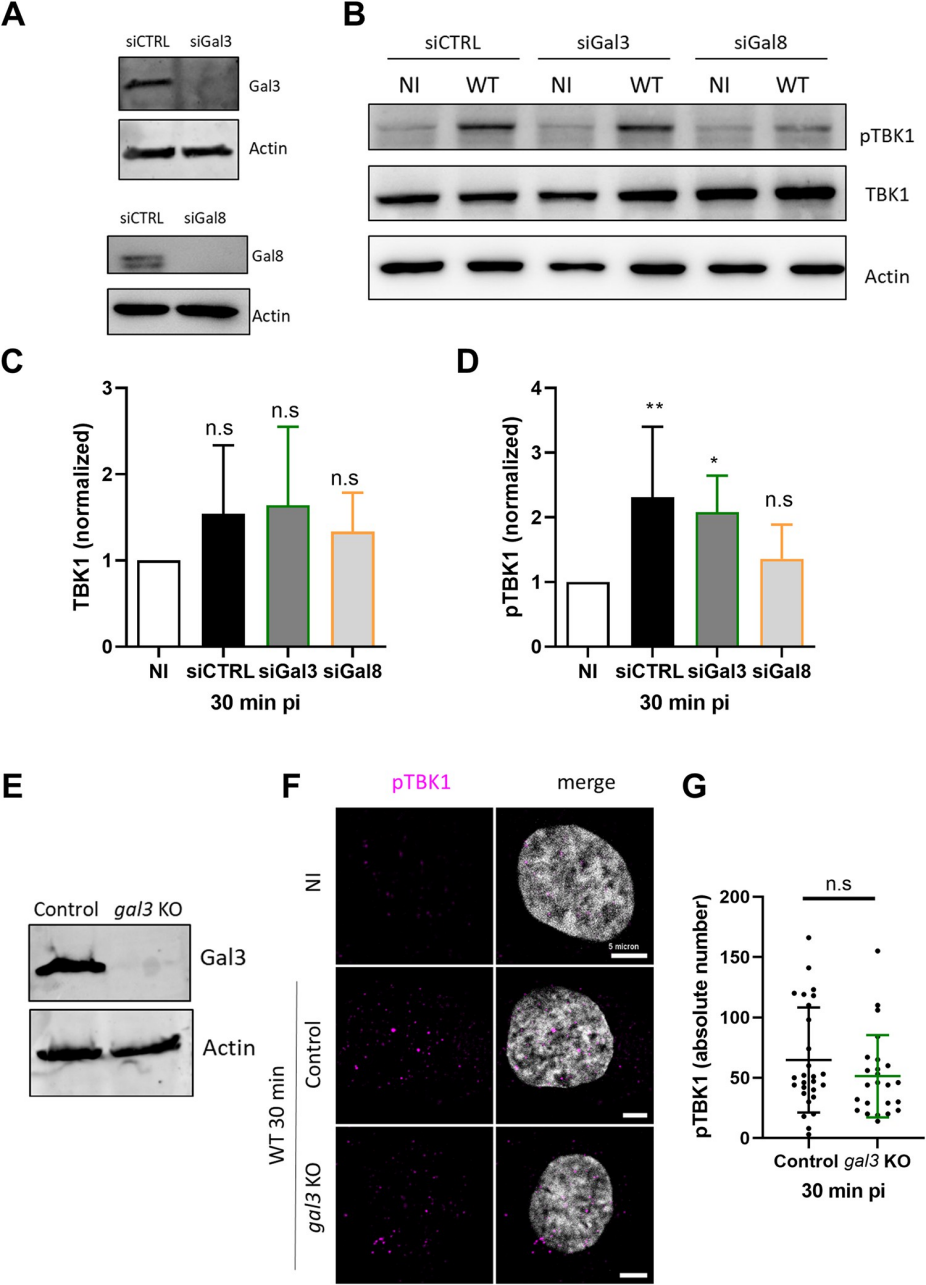
TBK1 activation involves Gal8 but not Gal3. (**A**) U2OS cells were transfected with a control siRNA (siCTRL) or siRNA targeting either Gal3 (siGal3) (**top panel**) or Gal8 (siGal8) (**bottom panel**) and analyzed by western blot using anti Gal3 and Gal8 antibodies, respectively. (**B**) U2OS control or depleted for Gal3 (green) or Gal8 (orange) cells were infected for 30 min with Ad WT and analyzed by western as in Fig 1A. Levels of TBK1 (**C**) and pTBK1 (**D**) were quantified for 3 independent experiments and normalized as in Fig 1B and Fig 1C, respectively. (**E**) U2OS and *gal3* KO cells were analyzed by western blot using anti Gal3 antibody. (**F**) U2OS and *gal3* KO cells were infected with Ad WT for 30 min and analyzed by immunofluorescence using anti pTBK1 antibody (magenta). A representative confocal image is shown. (**G**) Quantification of pTBK1 foci in U2OS control or *gal3* KO cells from F (n>30). P values are based on Ordinary ONE-WAY ANOVA analysis and Dunnett’s multiple comparison test for C and D and unpaired t-test for G.

**Fig 4:**
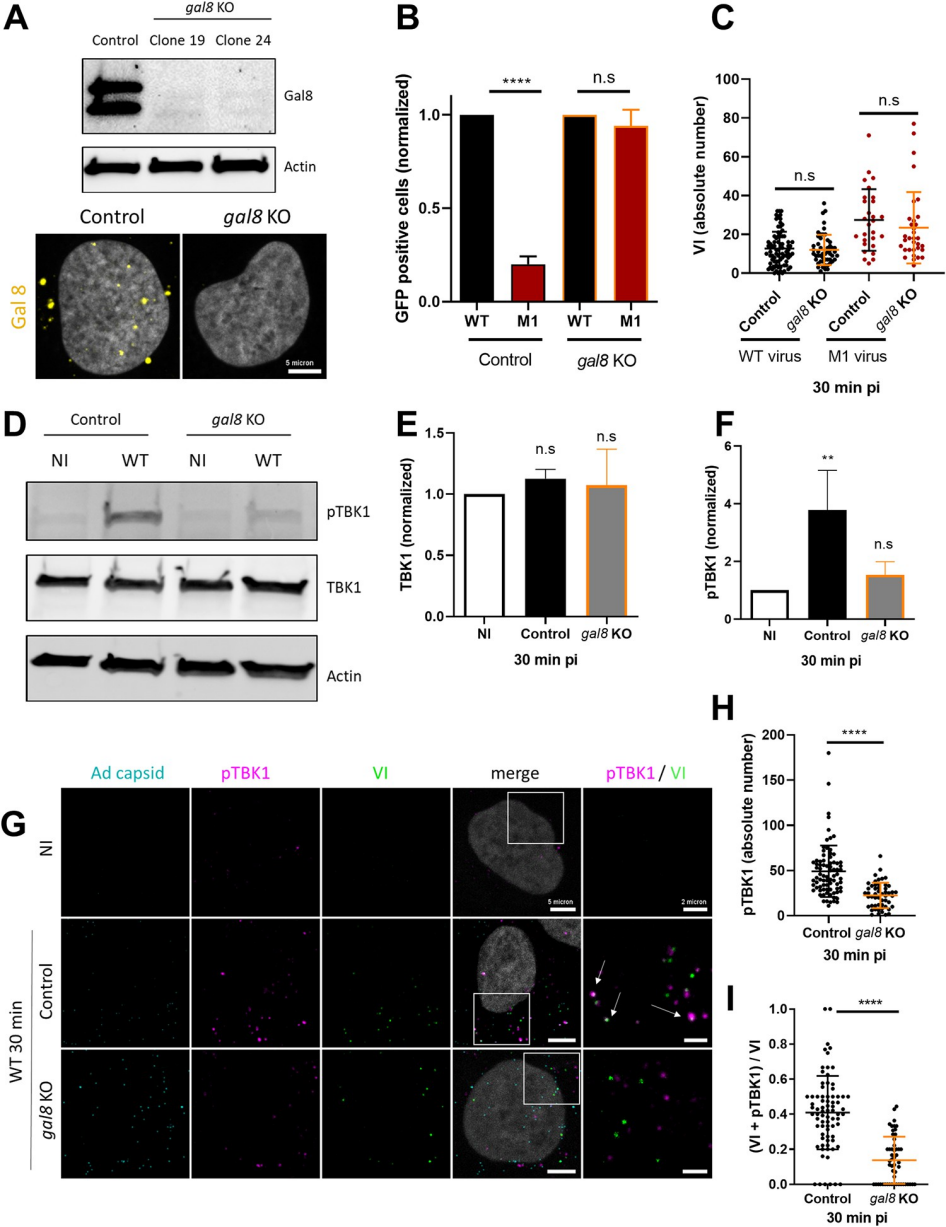
TBK1 activation and recruitment requires Gal8. (**A**) U2OS control or *gal8* KO cells were analyzed by western blot (**top panel**) and immunofluorescence (**bottom panel**) using anti Gal8 antibodies. (**B**) Control U2OS or *gal8* KO were infected with 50 pp/cell of Ad WT (black) or M1 (red), both expressing GFP. Percentage of GFP expressing cells was determined 24 h pi by flow cytometry. Data for Ad M1 were normalized to Ad WT. (**C**) Quantification of VI dots at 30 min pi for Ad WT (black), and M1 (red) in U2OS and *gal8* KO cells (n>30). (**D**) U2OS and *gal8* KO cells were infected with Ad WT for 30 min and analyzed by western blot as in Fig 1A. Levels of TBK1 (**E**) and pTBK1 (**F**) were quantified for 3 independent experiments and normalized as in Fig 1B and 1C, respectively. (**G**) U2OS control or *gal8* KO cells were infected with fluorescently labeled (cyan) Ad WT and analyzed at 30 min pi by immunofluorescence using anti protein VI (green) and anti pTBK1 (magenta) antibodies. Representative confocal images at 30 min pi are shown. An enlarged inset is shown to the right of each panel. White arrows indicate colocalizations between VI and pTBK1. (**H**) Quantification of pTBK1 foci in U2OS control or *gal8* KO cells (n>30). (**I**) Proportion of VI colocalization with pTBK1 at 30 min pi (n>30). P values are based on Ordinary ONE-WAY ANOVA analysis and Dunnett’s multiple comparison test for B, C, E, F and unpaired t-test for H and I.

The galectin-glycan system was previously shown to drive recruitment of TBK1 during bacterial invasion through the autophagy receptor NDP52 [13, 27]. Because we showed that Ad-induced membrane damage also recruits Gal3 and Gal8 [14], we next asked if either was needed for TBK1 activation. Both Gal3 and Gal8 were depleted from U2OS cells using specific siRNA, and depletion was confirmed by western blot (**Fig 3A**). The siRNA-treated U2OS cells were infected with Ad WT, and TBK1 activation was measured by western blot. Efficient TBK1 phosphorylation was detected in Ad-infected, but not in uninfected control cells. Gal3 silencing upon infection did not affect TBK1 phosphorylation while Gal8 silencing strongly reduced the TBK1 phosphorylation (**Fig 3B**). Quantification of western blots performed in triplicate showed that Gal3 or Gal8 depletion did not affect total TBK1 levels (**Fig 3C**). However, depletion of Gal8 (but not Gal3) efficiently prevented TBK1 phosphorylation upon infection (**Fig 3D**), suggesting that Gal8 was necessary for efficient TBK1 activation upon Ad entry. To confirm that the lack of effect after Gal3 depletion by siRNA is not due to residual Gal3 expression, we confirmed our results using U2OS-*gal3* KO cells line (hereafter *gal3* KO) generated by the CRISPR/Cas9 technology. The absence of Gal3 in *gal3* KO cells was confirmed by western blot (**Fig 3E**). We next stained pTBK1 in control and *gal3* KO cells infected for 30 min with Ad WT (**Fig 3F**). Quantification of pTBK1 foci showed no significant difference in the amount in control versus *gal3* KO cells (**Fig 3G**), confirming that Gal3 is not involved in TBK1 activation by Ad entry. To further address the role of Gal8 in TBK1 activation, we also generated U2OS-*gal8* KO cells (hereafter *gal8* KO) using CRISPR/Cas9 technology. The absence of Gal8 in *gal8* KO cells was confirmed by western blot and validated using immunofluorescence (**Fig 4A**). In addition we determined M1 infectivity, which is restricted by Gal8, in *gal8* KO cells [14]. We infected control cells and *gal8* KO cells with WT or M1 GFP expressing vectors, and determined GFP expression by flow cytometry the following day (**Fig 4B**). In control cells, M1 infectivity was reduced by ~80% compared to the WT. In contrast, in *gal8* KO cells M1 infectivity was restored to WT levels, reinforcing our previous report that identified Gal8 as a key restriction factor for the M1 virus [14]. WT infectivity was not affected by the absence of Gal8. To exclude that Gal8-mediated M1 infectivity restriction was linked to Ad endosome penetration efficiency, we quantified the numbers of VI foci (to mark endosome penetration sites) in U2OS control or *gal8* KO cells infected with either WT or M1 virus. The results showed that Gal8 did not influence VI release confirming that Gal8 is not required for Ad endosome lysis and viral escape *per se* (**Fig 4C**). We next re-analyzed TBK1 activation upon infection using control and *gal8* KO cells. As with the siRNA experiment, western blot analysis showed Gal8 dependence of TBK1 phosphorylation (**Fig 4D),** showing that absence of Gal8 impaired TBK1 phosphorylation without changing the total level of TBK1 (**Fig 4E and 4F**). Because our experiments indicated that Gal8 was necessary for TBK1 phosphorylation, we wondered if Gal8 was also necessary for pTBK1 recruitment to the site of membrane damage. Control U2OS cells or *gal8* KO cells were infected with Alexa-labeled Ad WT. Cells were fixed at 30 min pi and stained with specific antibodies against VI and pTBK1 (**Fig 4G**). The results showed that absence of Gal8 results in fewer pTBK1 foci than in control cells (**Fig 4H**) which were also less associated with Ad endosome penetration sites (**Fig 4I**). Together, these results show that Gal8 is central for TBK1 activation and recruitment towards the Ad membrane penetration site.

### TBK1 activation and recruitment to membrane penetration sites does not require SLR autophagy receptors

**Fig 5:**
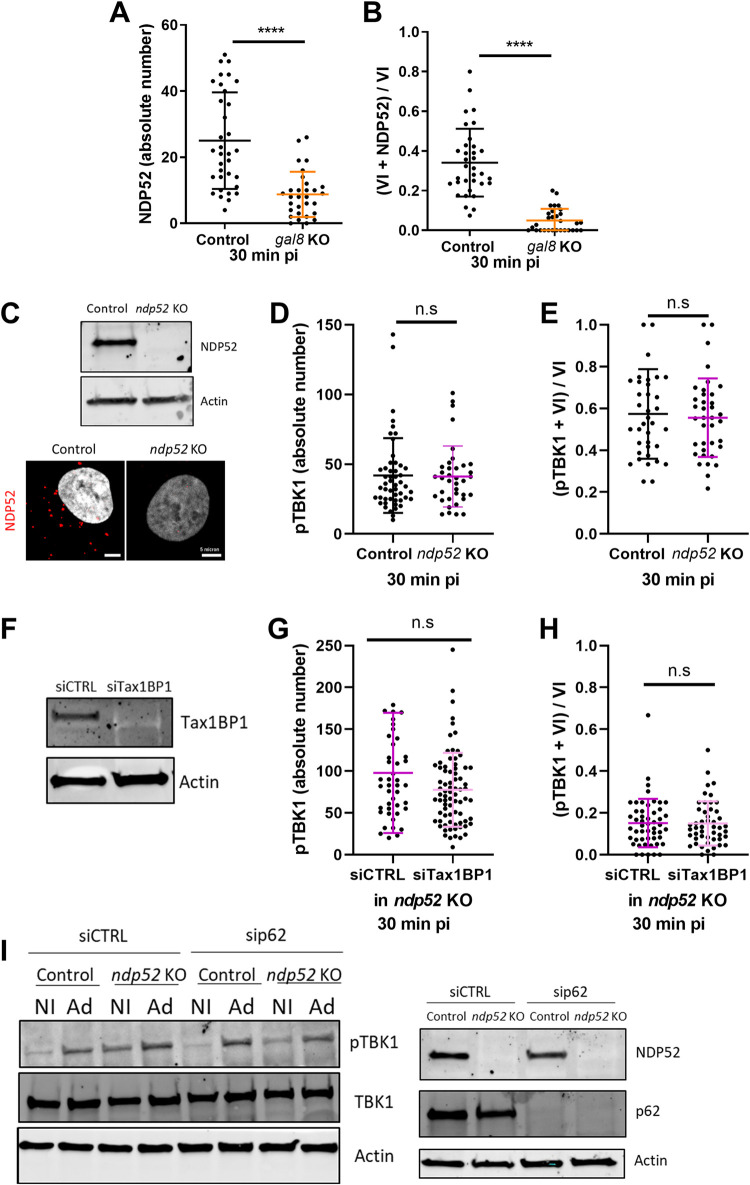
Conventional autophagic receptors are not involved in TBK1 recruitment or activation upon Ad infection. (**A**-**B**) U2OS control or *gal8* KO cells were infected with Ad WT and analyzed at 30 min pi by immunofluorescence using anti protein VI and anti NDP52 antibodies. Quantifications of NDP52 dots (**A)** and proportion of VI colocalization with NDP52 (**B**) are presented (n>30). (**C**) U2OS control and *ndp52* KO cells are analyzed by western blot (**top panel**) and immunofluorescence (**bottom panel**) using anti NDP52 antibody. (**D**-**E**) U2OS control or *ndp52* KO cells were infected with Ad WT and analyzed at 30 min pi by immunofluorescence using anti protein VI and anti pTBK1 antibodies. Quantification of pTBK1 foci (**D**) and proportion of VI colocalization with pTBK1(**E**) are shown (n>30). (**F**) U2OS *ndp52* KO cells were transfected with siCTRL or siRNA targeting Tax1BP1 (siTax1BP1) and analyzed by western blot using antibodies against Tax1BP1 and actin. (**G and H**) U2OS *ndp52* KO control or Tax1BP1 depleted cells were infected with Ad WT and analyzed at 30 min pi by immunofluorescence using anti protein VI and anti pTBK1 antibodies. Quantification of pTBK1 foci (**G**) and proportion of VI colocalization with pTBK1 (**H**) are shown (n>30). (**I**) U2OS control and *ndp52* KO cells were transfected with siCTRL or sip62, and then infected with Ad WT for 30 min. Cells were analyzed by western blot as in Fig 1A (**left panel**) and with anti NDP52, p62 and actin antibodies (**right panel**). P values are based on unpaired t-test.

In the *S*. *Typhimurium* bacterial model, autophagy receptors such as NDP52 may serve as assembly and/or activation hubs for TBK1 [27,30]. Autophagy receptors accumulate at the Ad penetration site [14]. We therefore asked whether NDP52 recruitment upon Ad penetration requires Gal8, since this could explain the accumulation of pTBK1. Immunofluorescence analysis was performed upon infection, and the total number of NDP52 dots, as well as their colocalization with VI, were quantified at 30 min pi. Quantification of NDP52 showed that in the absence of Gal8, significantly fewer NDP52 dots were detected (**Fig 5A**) and they rarely colocalized with VI signals (**Fig 5B**), indicating that NDP52 recruitment to Ad penetration sites is Gal8 dependent. This is compatible with a role for NDP52 in pTBK1 recruitment to the Ad penetration site. To test this, we generated U2OS-*ndp52* KO cells (hereafter *ndp52* KO) by CRISPR/Cas9 technology. Western blotting and immunofluorescence confirmed that NDP52 was absent from the KO cells (**Fig 5C**). To determine whether TBK1 activation and recruitment to the Ad penetration site require NDP52, we stained U2OS control cells and *ndp52* KO cells 30 min pi with antibodies against VI and pTBK1. There was no difference in the number of pTBK1 foci in control and *ndp52* KO cells, showing that NDP52 is not required for TBK1 activation (**Fig 5D**). To determine whether pTBK1 still accumulates at Ad penetration sites in the absence of NDP52, we quantified colocalization of VI and pTBK1 (**Fig 5E**). Again, no differences were seen, showing that NDP52 is not required for TBK1 activation or recruitment to Ad penetration sites. To verify that the NDP52 paralogue Tax1BP1 does not compensate for the loss of NDP52, we depleted both NDP52 and Tax1BP1 by transfecting *ndp52* KO cells with siRNA targeting Tax1BP1. Western blotting confirmed efficient Tax1BP1 depletion by the siRNA (**Fig 5F**). We counted the total number of pTBK1 dots (**Fig 5G**), and the number of pTBK1 dots at Ad penetration sites (**Fig 5H**), with and without Tax1BP1 co-depletion. This showed that even with combined depletion of NDP52 and Tax1BP1, TBK1 phosphorylation and recruitment still occurred upon Ad entry. We finally asked if p62, another SLR autophagy receptor that accumulates at Ad penetration sites [14], could be involved in TBK1 activation. We repeated the experiment above, this time depleting p62 with siRNA in *ndp52* KO cells. Western blotting confirmed efficient depletion of p62 (**Fig 5I, right panel**). TBK1 activation was monitored in WT infected cells at 30 min pi (**Fig 5I, left panel**). Western blotting showed that depletion of NDP52 and p62, both alone and in combination, did not prevent TBK1 activation upon Ad entry. Taken together, our data show that the autophagy receptors NDP52, Tax1BP1 and p62 are not required for activation or recruitment of TBK1 in response to Ad endosome penetration. These observations clearly distinguish TBK1 activation in the Ad model from that in the *Salmonella* model [27,30], see discussion for detail.

### Catalytically active TBK1 potentiates anti-adenoviral autophagy

**Fig 6:**
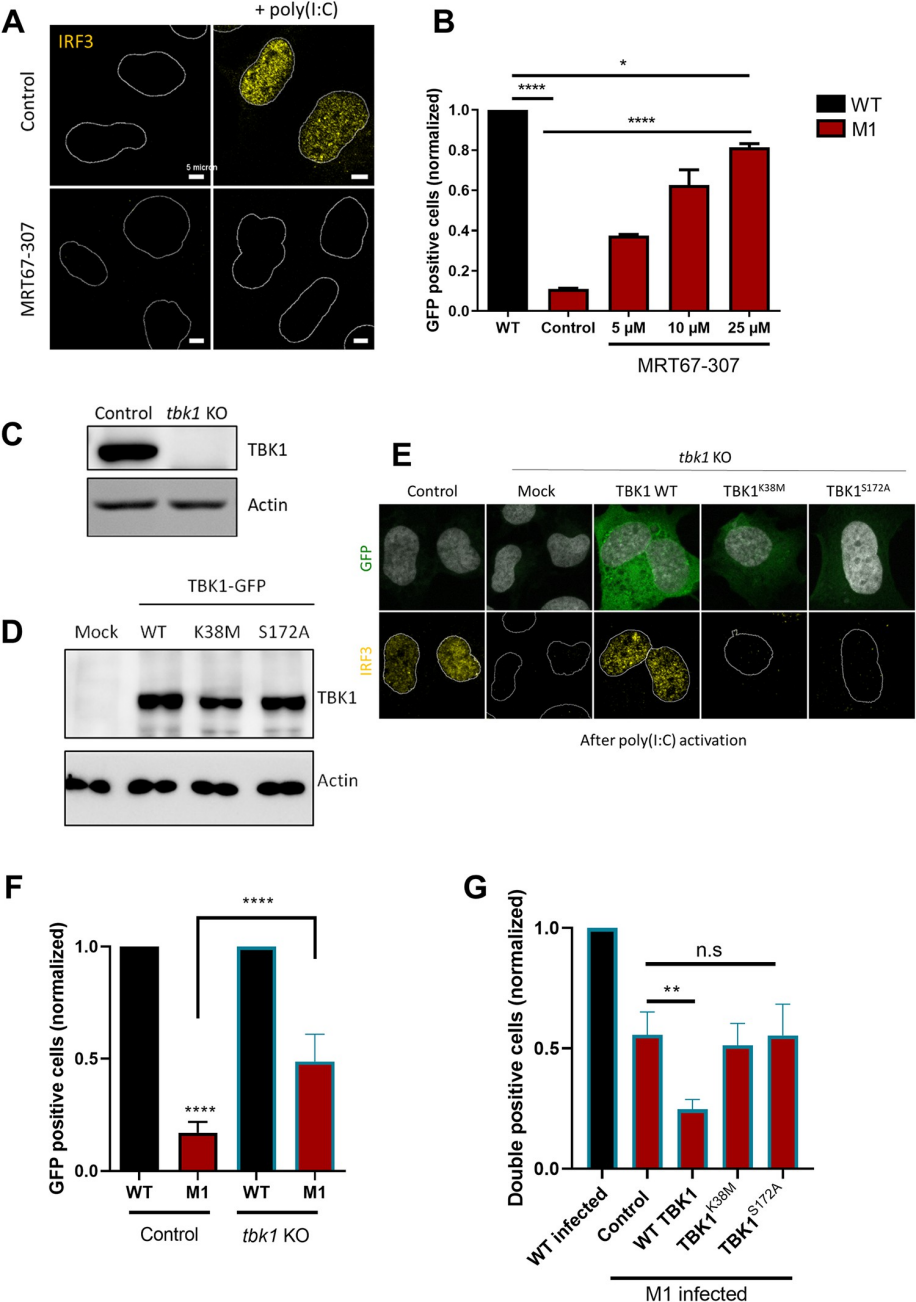
TBK1 kinase activity promotes autophagic degradation of Ad M1. (**A**) U2OS cells were pre-treated with vehicle (**top panel**) or with 5 μM of MRT67-307 (**bottom panel**) for 3 h and transfected with poly(I:C) for 3 h (**right panel**). Cells were analyzed by immunofluorescence using antibodies against IRF3 (yellow). Representative confocal images are shown and the nucleus is marked with a white line. (**B**) U2OS cells were pre-treated for 3 h with vehicle (control) or different concentrations of MRT67-307 and infected with 50 pp/cell of Ad WT (black) or M1 (red), both expressing GFP. Percentage of GFP expressing cells was determined 24 h pi by flow cytometry and normalized to Ad WT. (**C**) U2OS control or *tbk1* KO cells were analyzed by western blot using anti TBK1 antibody. (**D**) *tbk1* KO cells were transfected with plasmid coding for GFP-tagged TBK1 WT or mutant (TBK1^K38M^ or TBK1^S172A^) and analyzed by western blot using antibodies against TBK1. (**E**) *tbk1* KO cells were transfected by GFP-tagged TBK1 plasmids followed by poly(I:C) transfection as in D and analyzed by immunofluorescence using IRF3 antibody (yellow). The nucleus is marked with a white line. (**F**) U2OS control or *tbk1* KO cells were infected with 50 pp/cell of WT (black) or M1 (red), both expressing GFP. The percentage of GFP expressing cells was determined 24 h pi by flow cytometry and normalized to Ad WT. (**G**) *tbk1* KO cells were transfected with control or GFP-tagged TBK1 plasmids and infected with 10 pp/cell of mCherry Ad WT or M1. The percentage of GFP and mCherry expressing cells was determined by flow cytometry and normalized to Ad WT. P values are based on Ordinary ONE-WAY ANOVA analysis and Dunnett’s multiple comparison test.

The unexpected autonomy of TBK1 recruitment to the site of Ad membrane penetration from different autophagy receptors suggested that two functionally independent complexes could be activated upon Ad infection. One Gal8 and TBK1 containing complex involved in sensing the membrane damage and a second complex containing SLRs and mediating autophagy activation. To understand the placing of TBK1 in this relationship, we next used inhibitor MRT67-307 [38] to alter TBK1 function during Ad infection. We used IRF3 (Interferon Regulatory Factor 3) localization as a marker for TBK1 activity. IRF3 is a transcription factor and cytosolic target of active TBK1 that, once phosphorylated, translocates into the nucleus where it drives an antiviral response [39]. U2OS cells were pre-treated for 3 h with 5 μM of MRT67-307 or vehicle and transfected with poly(I:C), a known inducer of the TBK1/IRF3 pathway [40]. In non-stimulated cells, IRF3 is cytosolic (**Fig 6A, left panel**), while poly(I:C) treatment resulted in nuclear translocation of IRF3, which was blocked in MRT67-307 treated cells (**Fig 6A, right panel**). We next pre-treated U2OS cells for 3 h with increasing amounts of MRT67-307, and infected treated cells for 24 h with WT or M1 vectors expressing GFP. The number of GFP positive cells was quantified by flow cytometry and M1 infectivity was normalized to the WT (**Fig 6B**). In control cells, M1 infectivity was ~10 fold reduced compared to the WT, due to autophagic degradation [14]. With increasing amount of MRT67-307, M1 infectivity was partially and selectively rescued in a dose-dependent manner, suggesting that TBK1 inhibition progressively inhibited autophagy, thus preventing M1 degradation. However, MRT67-307 is not specific for TBK1 inhibition, but is also know to alter other kinase activities including IKKε and ULK1 [41].

To independently determine the function of TBK1 in driving autophagy, we engineered U2OS-*tbk1* KO cells (hereafter *tbk1* KO) using CRISPR/Cas9 technology. We confirmed TBK1 removal by western blot (**Fig 6C**) and functionally validated the *tbk1* KO by showing that no IRF3 nuclear translocation was detected after poly(I:C) transfection (**Fig 6E, second column**). We repeated the infection experiment comparing WT and M1 infectivity in *tbk1* KO cells versus control cells. The analysis showed that M1 infectivity is specifically increased upon *tbk1* KO while WT infectivity remained unchanged; although the M1 rescue was only partial (**Fig 6F**). To confirm that the M1 infectivity gain was directly caused by the absence of TBK1, we next reconstituted the *tbk1* KO cells with wild type TBK1 and TBK1 mutants. Cells were transfected with vectors expressing GFP-fused to catalytically active TBK1 (WT TBK1) or two mutants encompassing a dead kinase domain (TBK1^K38M^) or the phosphorylation mutant (TBK1^S172A^) to study if enzymatic activity and/or activation by phosphorylation are required for restricting the M1 mutant infectivity. We verified equal TBK1 expression levels upon transfection by western blot using specific antibodies (**Fig 6D**) and confirmed that trans-complementation with TBK1 WT is functional by rescuing IRF3 nuclear translocation in *tbk1* KO cells, while the TBK1 mutants were functionally impaired (**Fig 6E**). To determine the effect of wild type and mutated TBK1 versions, *tbk1* KO cells transfected with TBK1 expression plasmids (GFP positive) were infected with WT or M1 vector expressing mCherry as transgene (mCherry positive) and the percentage of double positive fluorescent cells was quantified 24 h pi by flow cytometry and normalized to the WT vector (**Fig 6G**). Our results show that only re-introduction of WT TBK1 into *tbk1* KO cells reduced M1 infectivity showing that a catalytically active form of TBK1 is required for infectivity restriction of the autophagy sensitive M1 mutant virus. However, the restriction was less stringent than with the MRT67-307 inhibitor suggesting that other kinases (e.g. ULK1) may also contribute.

### TBK1 is central to autophagy activation upon Ad membrane penetration

**Fig 7:**
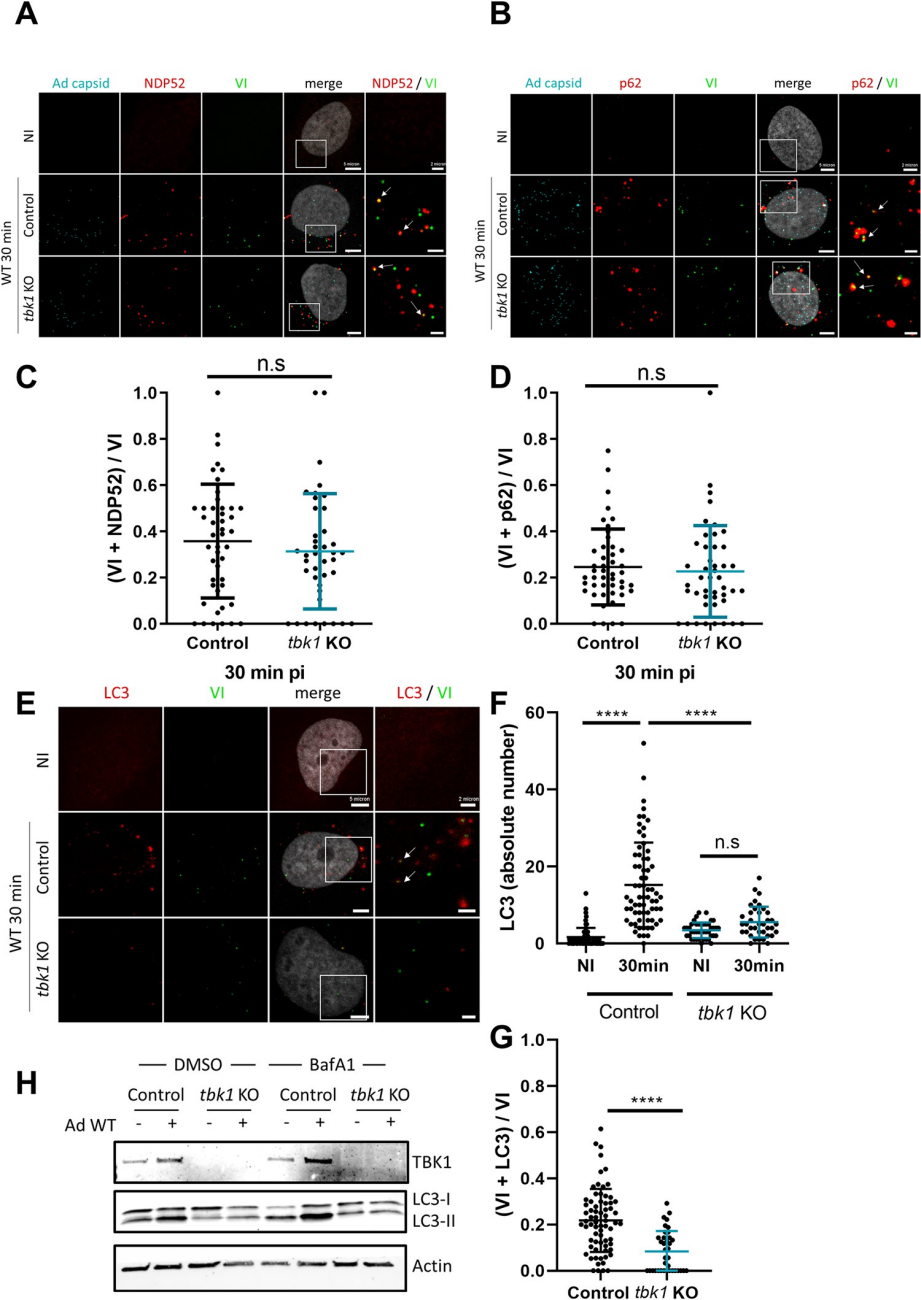
TBK1 is not involved in autophagic receptor recruitment to Ad penetration site. (**A**) U2OS control or *tbk1* KO cells were infected with fluorescently labeled Ad WT (cyan) and analyzed by immunofluorescence at 30 min pi using anti protein VI (green) and anti NDP52 (red) antibodies. Representative confocal images are shown. An enlarged inset is shown to the right of each panel. White arrows indicate VI and NDP52 colocalizations. (**B**) U2OS control or *tbk1* KO cells were infected with fluorescently labeled Ad WT (cyan) and analyzed by immunofluorescence at 30 min pi using anti protein VI (green) and anti p62 (red) antibodies. Representative confocal images are shown with enlarged insets to the right of each panel. White arrows indicate VI and p62 colocalizations. (**C**) Proportion of VI colocalization with NDP52 (n>30). (**D**) Proportion of VI colocalization with p62 (n>30). (**E**) U2OS control or *tbk1* KO cells were infected with Ad WT and analyzed by immunofluorescence at 30 min pi using anti protein VI (green) and anti LC3 (red) antibodies. Representative confocal images and enlarged insets to the right on each panel are shown. (**F**) Quantification of LC3 dots from E (n>30). (**G**) Proportion of VI colocalization with LC3 (n>30). (**H**) U2OS control or *tbk1* KO cells were pre-treated for 3 h with Bafilomycin A1 (BafA1) or DMSO. Cells were then infected with Ad WT for 45 min, and analyzed by western blot using anti TBK1, LC3 and actin antibodies. P values are based on Ordinary ONE-WAY ANOVA analysis and Dunnett’s multiple comparison test for F and unpaired t-test for C, D and G.

After demonstrating that TBK1 controls M1 infectivity through its kinase activity, we next wanted to show that this is executed via autophagy activation. TBK1 phosphorylates several autophagy receptors in anti-bacterial autophagy, enhancing their recruitment to the membrane damage site [27,29–31]. To address if TBK1 also affects NDP52 or p62 recruitment in adenoviral membrane penetration, we performed immunofluorescence analysis using *tbk1* KO cells infected with WT virus. We analyzed colocalization between viral protein VI and NDP52, or p62, at 30 min pi. Both NDP52 (**Fig 7A**) and p62 (**Fig 7B**) colocalize with membrane penetration sites frequently in both *tbk1* KO and control cells (shown by arrows). Quantifying the colocalization revealed that NDP52 (**Fig 7C**) and p62 (**Fig 7D**) are equally present at Ad penetration sites whether TBK1 is present or not. This shows that TBK1 does not control initial recruitment of either receptor. To clarify if TBK1 nevertheless plays a role in subsequent Ad-induced autophagy, we used LC3 lipidation (LC3-II) as a marker for autophagy activation. We first quantified LC3 punctae by immunofluorescence, indicating autophagosome formation upon Ad infection, in presence or absence of TBK1 (**Fig 7E**). In agreement with previous observations at 30 min pi, LC3 punctae accumulated in infected control cells [14]. In contrast, much less LC3 punctae were formed upon infection in *tbk1* KO cells (**Fig 7F**), resulting in decreased accumulation of LC3 at Ad penetration sites (**Fig 7G**). We next infected control or *tbk1* KO cells for 45 min with Ad WT and analyzed the LC3 lipidation status by western blot (**Fig 7H**). LC3 lipidation upon autophagy activation results in a small size shift in western blot analysis [42]. We observed LC3 lipidation upon Ad infection in control cells but almost none in *tbk1* KO cells. This difference was even more pronounced when cells were treated with Bafilomycin A1 to block autophagosome maturation resulting in accumulation of newly formed autophagosomes. Together, these results suggested that anti-adenoviral autophagy is severely limited in the absence of TBK1, highlighting a central function for TBK1 in driving autophagy in response to Ad membrane penetration.

**Fig 8:**
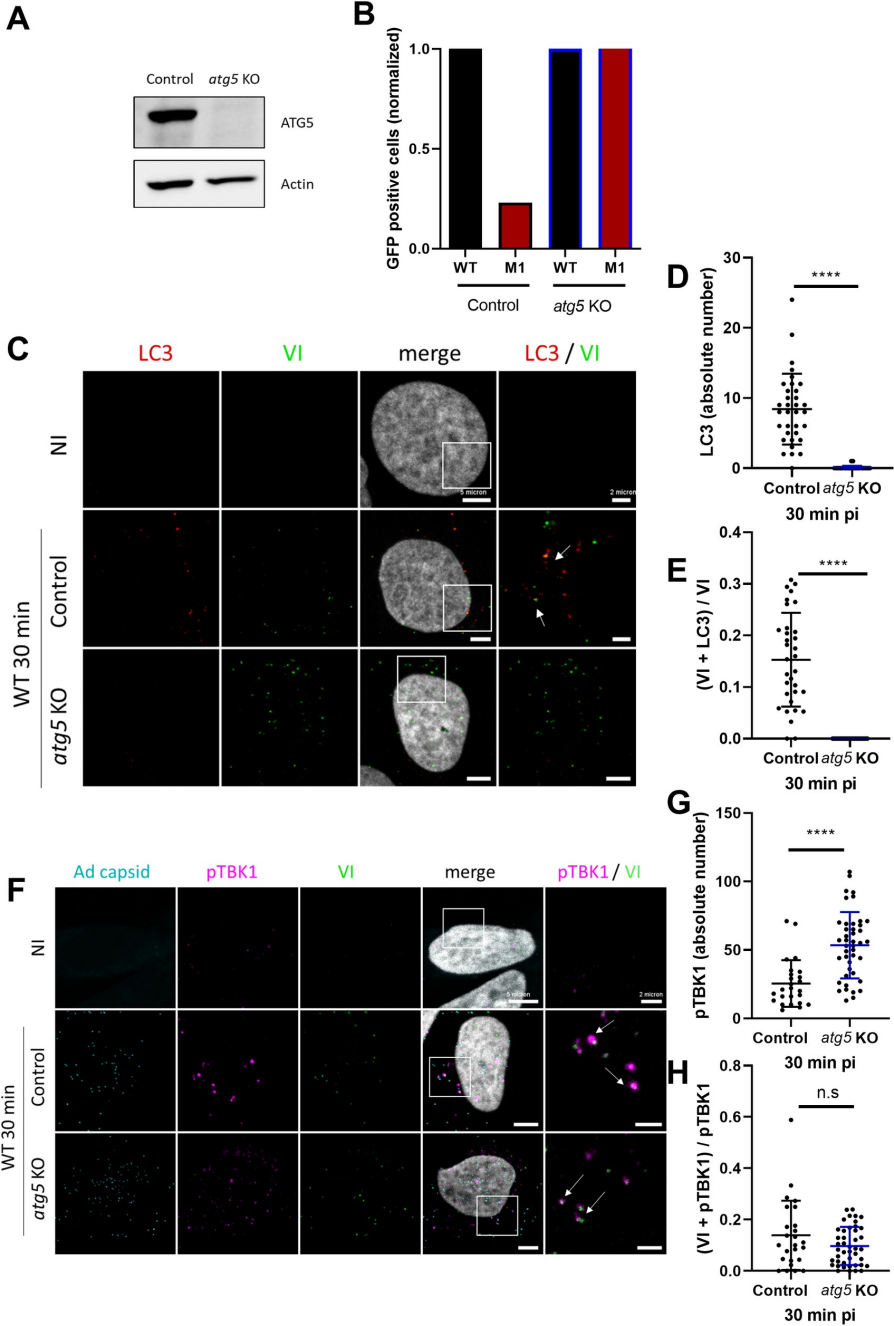
ATG5 function is not required for TBK1 recruitment. (**A**) U2OS control or *atg5* KO cells were analyzed by western blot using anti ATG5 antibody. (**B**) U2OS control or *atg5* KO cells were infected with 50 pp/cell of Ad WT (black) or M1 (red), both expressing GFP. Percentage of GFP expressing cells was determined 24 h pi by flow cytometry and normalized to Ad WT. (**C**) U2OS control and *atg5* KO cells were infected with Ad WT and analyzed at 30 min pi by immunofluorescence using anti protein VI (green) and anti LC3 (red) antibodies. Representative confocal images and enlarged insets to the right of each panel are shown. White arrows represent VI and LC3 colocalizations. (**D**-**E**) Quantification of LC3 dots (n>30) (**D**) and proportion of VI colocalization with LC3 (n>30) (**E**). (**F**) U2OS control and *atg5* KO cells were infected with fluorescently labeled Ad WT (cyan) and analyzed at 30 min pi by immunofluorescence using anti protein VI (green) and anti pTBK1 (magenta) antibodies. Representative confocal images and enlarged insets to the right of each panel are shown. White arrows represent VI and pTBK1 colocalizations. (**G**-**H**) Quantification of pTBK1 dots (n>30) (**G**) and proportion of VI colocalization with pTBK1 (n>30) (**H**). P values are based on unpaired t-test.

To understand if TBK1 is recruited to membrane penetration sites prior or as part of the autophagy response, we removed key autophagy components. As part of the LC3 lipidation machinery, ATG5 is required for autophagosome elongation [43]. Moreover, ATG5 may participate in a negative feedback during TBK1 activation by inducing its autophagic degradation [44]. Here, we engineered U2OS-*atg5* KO (hereafter *atg5* KO) using CRISPR/Cas9 and validated them by western blot (**Fig 8A**) and through complete infectivity restoration of the autophagy sensitive M1 virus (**Fig 8B**) [14]. As expected, no LC3 dots were detected upon Ad infection in *atg5* KO cells (**Fig 8C and 8D**), showing that autophagy mounted at Ad penetration sites requires ATG5 (**Fig 8E**). We next quantified TBK1 activation and recruitment to Ad penetration sites in the absence or presence of ATG5. Control cells or *atg5* KO cells were infected with Ad WT and analyzed by immunofluorescence 30 min pi (**Fig 8F**). Quantification of pTBK1 foci showed that they accumulated to an even larger extent in infected *atg5* KO cells than in infected U2OS control cells (**Fig 8G**). To account for this difference, we normalized pTBK1 colocalizing with protein VI to the total number of pTBK1 foci detected, to estimate the fraction of pTBK1 present at Ad penetration sites in each cell line (**Fig 8H**). The normalization confirmed that the absence of ATG5 (i.e. autophagy) neither prevents nor alters pTBK1 recruitment to virus-induced membrane damage sites. Furthermore, this shows that TBK1 acts upstream of autophagy activation and not as its consequence. Absence of autophagy may also account for the increased overall number of pTBK1 foci supporting the observation that autophagy limits TBK1 activation in a negative feedback loop [44].

### pTBK1 preferentially responds to Ad-induced membrane damage

**Fig 9:**
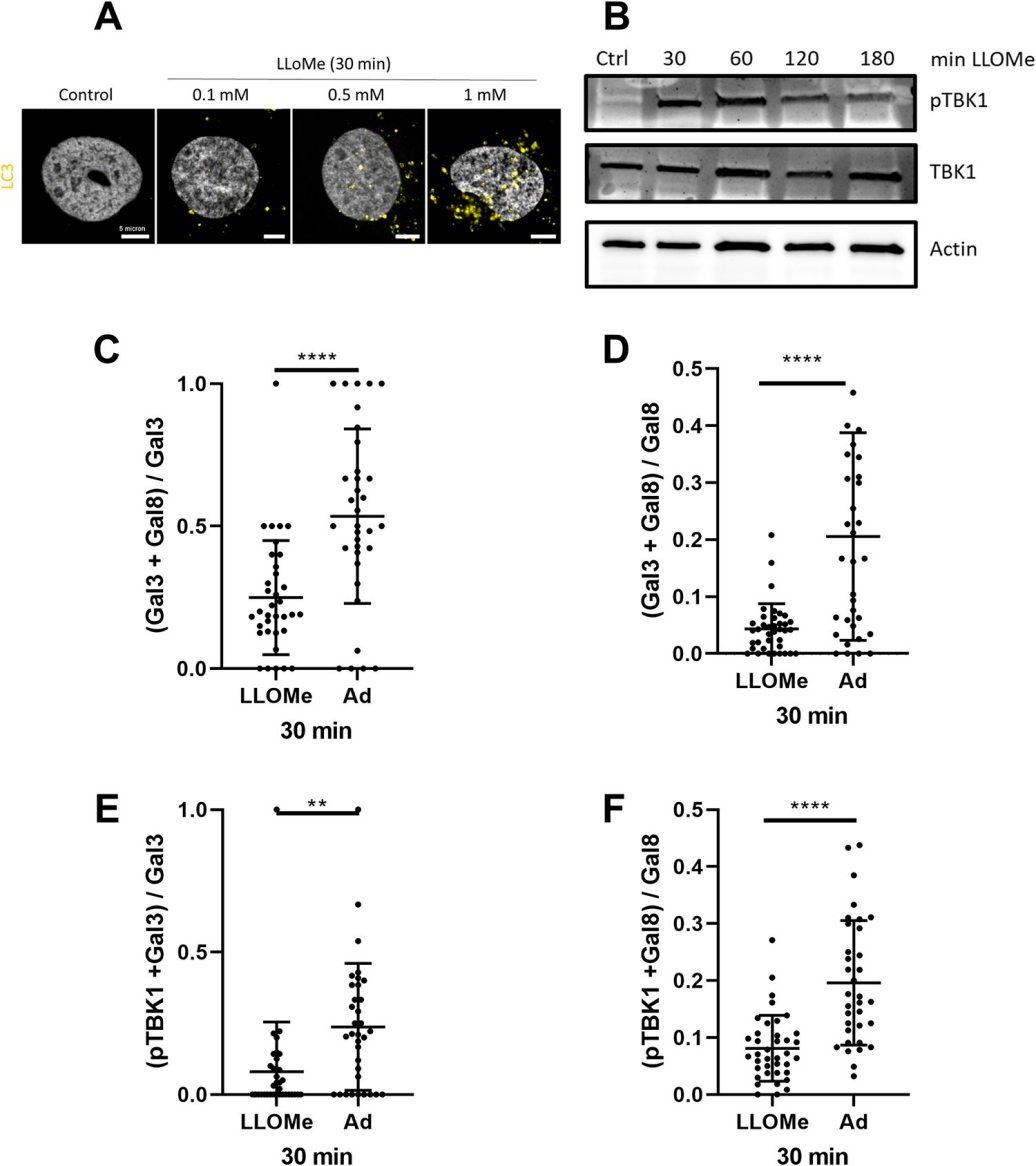
Distinct damaged membranes detection relies on TBK1 activation and recruitment. (**A**) U2OS cells were treated for 30 min with increasing amounts of LLOMe (0.1 mM to 1 mM). Immunofluorescence was performed using anti LC3 antibody (yellow) and representative confocal images are shown. (**B**) U2OS cells were treated with 1 mM of LLoMe for different times (30 min to 180 min) and analyzed by western blot as in Fig 1A. (**C**-**F**) U2OS cells were infected with Ad WT and analyzed at 30 min pi by immunofluorescence using anti pTBK1, anti Gal3 or anti Gal8 antibodies. Quantification of Gal3 and Gal8 colocalizations were normalized to Gal3 (**C**) or to Gal8 (**D**) (n>30). Quantification of pTBK1 and Gal3 (**E**) or pTBK1 and Gal8 (**F**) normalized to Gal3 and Gal8, respectively (n>30). P values are based on unpaired t-test.

Our data indicate that Gal8 and TBK1 are part of an autophagy-independent damage sensing complex. We wondered if this is specific to Ad or if Gal8 and TBK1 are part of a wider machinery sensing membrane damage. To address this question, we treated cells with LLOMe, a specific inducer of sterile lysosomal membrane damage [45]. We first showed that 1 mM LLOMe induces LC3 lipidation and activates autophagy (**Fig 9A**). U2OS cells were treated with LLOMe for different times, and TBK1 phosphorylation was followed by western blotting (**Fig 9B**). TBK1 phosphorylation was detectable 30 min after LLOMe treatment and maintained over time. This is consistent with a recent report that TBK1 phosphorylation is detectable 1 h after LLOMe treatment [26]. We next analyzed if pTBK1 is recruited to sites of membrane damage induced by LLOMe. Gal3 and Gal8 are often used to detect membrane damage, including lysosome damage, and pTBK1 was shown to colocalize with Gal3 upon LLOMe treatment[46]. However, to the best of our knowledge, it is not known if Gal3 and Gal8 detect membrane damage equally or if they have distinct roles. Thus, we started by comparing Gal3 and Gal8 recruitment to sites of lysosomal damage induced by LLOMe and sites of early endosome damage induced by Ad WT. U2OS cells were treated for 30 min with 1 mM LLOMe or infected with Ad WT, then stained for Gal3 and Gal8. Gal3 and Gal8 colocalization was quantified and normalized to total Gal3 (**Fig 9C**) or Gal8 (**Fig 9D**). Colocalization was induced by both LLOMe and Ad. The proportion of Gal3 colocalizing with Gal8 (**Fig 9C**) was much higher than that of Gal8 colocalizing with Gal3 (**Fig 9D**), suggesting preferential co-recruitment of Gal3 to Gal8 positive sites and not the other way round. Colocalization of Gal3 and Gal8 was more often seen at sites of Ad-induced membrane damage (~50%) than at sites of LLOMe-induced damage (~25%). We conclude that there are indeed differences in the sensing of damaged endosomal and lysosomal membranes, with detection by both galectins preferentially occurring on endosomes damaged by Ad. We next quantified if pTBK1 colocalizes more specifically with one of the galectins and if this depends on the damaged compartment (**Fig 9E and 9F**). The results show that pTBK1 colocalizes with both galectins. Due to the high colocalization ratio, we could not determine whether a specific galectin was responsible. However, pTBK1 was more commonly recruited to galectins at sites of early endosomal membrane damage caused by Ads, than to lysosomal membrane damage sites caused by LLOMe. This suggests that galectin density and/or alterations in the composition of the damaged membrane influence pTBK1 recruitment. We conclude that TBK1 is part of a sensing machinery that uses galectins to detect a wide range of membrane damage. It is thus well placed for fast and efficient detection of invading viruses.

## Discussion

Translocating across the cell membrane without fusion is a hallmark of non-enveloped virus entry, a process that relies on membrane modification or rupture [15,47–51]. Given our detailed understanding of the adenoviral life cycle, Ads are ideal tools to study membrane penetration by non-enveloped viruses. Here we investigated the cellular response to membrane penetration by Ads and asked whether it differs from the cellular response to membrane damage caused by bacteria. By monitoring membrane integrity and detecting membrane damage or distortion, cells seize a golden opportunity to catch invaders early in infection. Initially described for the invasive bacterium *S*. *typhimurium*, membrane rupture activates a protective autophagic response to degrade the pathogen, eliminate the associated membrane damage, and restore cellular homeostasis [13,27,30]. This principle has subsequently been shown to apply to several, if not all invasive bacterial species [52–54]. Bacteria are relatively large pathogens, causing extensive membrane damage, elicited often from specific, bacteria-containing vacuoles. In addition, bacteria produce an arsenal of effector molecules to divert and counteract the cellular response to membrane damage [55–58]. In comparison, virus-induced membrane damage is rather subtle; takes place in a conventional cellular organelle, the endo-lysosomal compartment; and must be counteracted by proteins already present in the entering virion.

**Fig 10:**
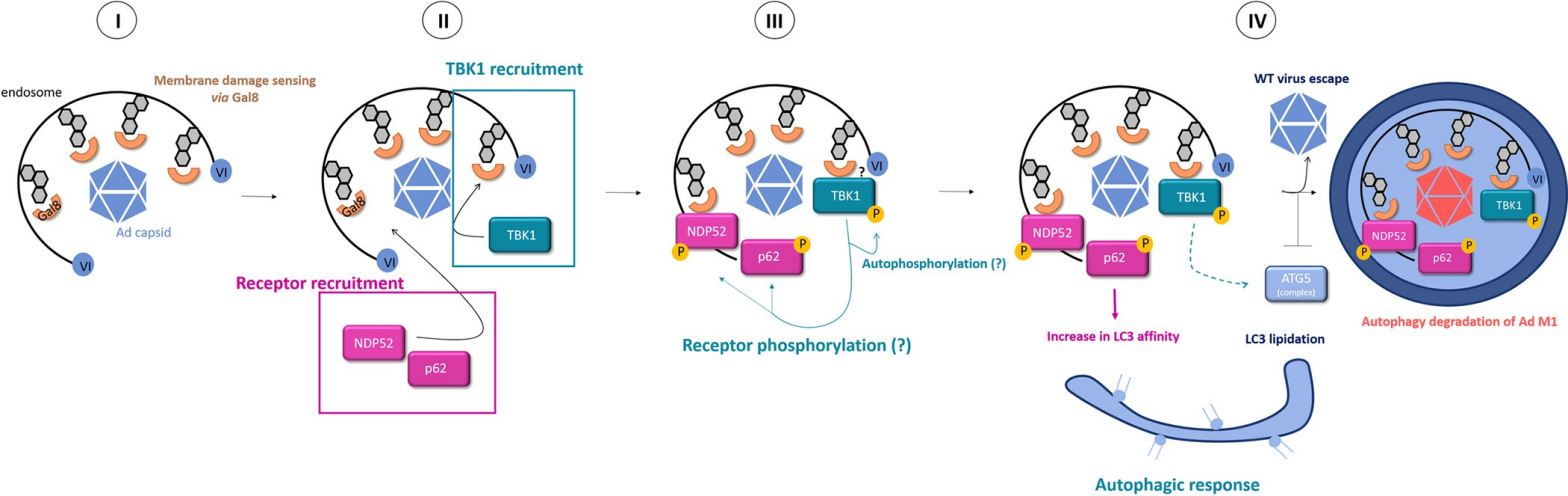
Model of autophagy activation via TBK1 at Ad endosome penetration sites. Ad entry induces rupture of the endosomal membrane exposing intracellular glycans which are detected by Gal8 (**I**). This step creates a recruitment and activation hub for TBK1 to the Ad penetration site. Autophagy receptors (NDP52, p62) are recruited independently of TBK1 (**II**). TBK1 recruitment results in high local concentrations at the penetration site probably leading to its activation by auto-phosphorylation. Once activated, TBK1 promotes autophagy activation as well as phosphorylates autophagy receptors increasing their affinity to LC3 (**III**). Autophagy activation generates autophagosomal membranes with LC3 and autophagy receptor link damaged membranes into the forming autophagosome to induce degradation of the damaged membrane (including associated pathogens, i.e. Ad M1) to restore homeostasis (**IV**). In contrast, Ad WT stalls autophagosome formation and/or maturation and avoids degradation by escaping into the cytosol.

We show that Ad-induced membrane damage induces TBK1 phosphorylation on S172. TBK1 activation is not linked to upstream events such as receptor binding or endocytosis because the endocytosed, but non-membrane lytic, TS1 mutant failed to activate TBK1. Activated TBK1 was localized to the Ad penetration site, suggesting that it might functionally link viral penetration to the downstream cellular response. TBK1 activation occurs during bacterial invasion [33] and is required to control bacterial proliferation [27]. Thurston and colleagues showed that catalytically inactive TBK1 can be found in association with intracellular *S*. *typhimurium* and that functional TBK1 recruitment to invading bacteria restricts their proliferation [27]. For Group A *Streptococcus*, it was shown that endogenous pTBK1 and bacteria colocalize under conditions that restrict bacterial growth [33]. We show that the recruitment of pTBK1 to Ad penetration sites depends on the presence of the cytosolic lectin Gal8 (**Fig 10, part I**). The Gal8 dependence for pTBK1 recruitment is highly specific because another recruited galectin, Gal3, failed to recruit pTBK1 in cells lacking Gal8. Furthermore, the timing of activation of TBK1 and its recruitment to the penetration site within 20 min of infection coincides with the detection of the viral membrane damage by galectins. In *S*. *typhimurium* infections, the recruitment of TBK1 to bacterially-damaged vacuoles requires its interaction with NAP1/Sinbad adaptors that, in turn, interact with the autophagy receptor NDP52 [27]. The adaptors and NDP52 link TBK1 to the Gal8-decorated ruptured vacuole surrounding the bacteria [59]. Forced recruitment of TBK1 to sites of membrane damage by direct fusion to Gal8 restricts bacterial proliferation. Consequently, direct fusion of TBK1 with NDP52 bypassed NAP1/Sinbad resulting in the same anti-proliferative effect on bacterial invasion [27]. The authors conclude that the early recruitment of TBK1 to the bacterial invasion site requires NDP52 and Gal8. TBK1 was also shown to functionally interact with other SLRs, such as p62, optineurin and Tax1BP1, resulting in their phosphorylation[28,29,31]. Like NDP52, these SLRs can be recruited to bacterial invasion sites, and phosphorylation of optineurin by TBK1 was shown to control *Salmonella* growth [28]. Despite these observations, the exact mechanism whereby TBK1 is initially recruited to the membrane damage site remains unclear. Efficient clustering of SLRs takes place during Ad endosome penetration [14]. However, we show that deletion of NDP52 alone or in combination with p62 depletion did not limit TBK1 activation upon Ad entry, nor did the deletion of NDP52 alone or in combination with Tax1BP1 impact the clustering of pTBK1 at the penetration site. Consequently SLRs, in contrast to Gal8, neither determine recruitment nor phosphorylation of TBK1 during Ad penetration (**Fig 10, part II**). Absence of TBK1 in turn did not prevent recruitment of p62 or NDP52 to the Ad penetration site. This observation implies that SLRs (i.e. NDP52) and TBK1 are recruited independently to the Ad penetration site, but both require Gal8. Independent recruitment has also been suggested recently in the context of bacterial membrane damage [60]. Receptors other than SLRs might thus be involved in TBK1 recruitment, for example TBC1D9 [33] or TRIM23 [61].

TBK1 regulates autophagy activation during bacterial invasion [27,29,59]. Our previous results showed that Ad penetration of the endosomal membrane also results in autophagy activation and that Ad stalls autophagy through a small capsid encoded PPxY peptide motif in protein VI to secure endosomal escape. Mutating the peptide motif, as in the M1 mutant, renders the virus fully susceptible to autophagic degradation. This property means that M1 infection can be used to probe the effectiveness of the cellular autophagic response [14,35]. Our results showed that Ad membrane penetration by both the WT and M1 viruses triggers TBK1 phosphorylation. TBK1 phosphorylation occurs either by trans-autophosphorylation [36] or via kinases involved in autophagy activation, such as AMPK [62]. The Gal8 dependence of TBK1 activation may indicate that TBK1 recruitment to Ad penetration sites results in local increased concentration thereby promoting oligomerization and trans-autophosphorylation. We observed that TBK1 activation upon entry is transient and cell type independent whether cells were infected with vector particles or replication competent virus particles (not shown) suggesting regulation at the endosome prior to viral gene expression. If limiting TBK1 activation is an active virus-driven process or an indirect consequence of accelerated escape remains to be shown. Deletion or pharmacological inhibition of TBK1 specifically rescued M1 infectivity without affecting WT infectivity showing that TBK1 impairment under Ad infection conditions prevents efficient autophagy induction. We did not investigate what substrate TBK1 phosphorylates, if any, during Ad-induced autophagy.

TBK1 can phosphorylate a range of SLRs in bacterial models for example, optineurin for Salmonella [28] and p62 for Mycobacteria [32].This potentiates the antibacterial response by stabilizing the autophagic complexes around the vacuole containing the bacteria. The ability of TBK1 to enhance autophagy via its kinase activity is also seen in mitophagy [24,29,63]. Thus, a similar role for TBK1 in SLR phosphorylation during Ad entry is possible (**Fig 10, part III**). However, we find a less prominent role for SLRs in our viral model, which could reflect differences in the extent of damage or in the composition of the organelle from which the pathogen escapes. For example, bacterial vacuoles containing *S*. *pneumoniae* sequentially recruit NDP52 and p62 concomitantly with vacuole maturation and autophagy activation [64]. In addition, other TBK1 substrates are known to play a role in autophagy regulation [65,66] including the promotion of WIPI2 membrane association to mediate assembly of the ATG5 complex [30]. Our infection studies in autophagy-deficient KO cells clearly place TBK1 upstream of autophagy activation in the cellular response to Ad infection. This is an important observation because it shows that TBK1 has a dual function participating initially in membrane damage sensing and subsequently in driving the ensuing autophagic response (**Fig 10, part IV**). Our data demonstrate that this sensor/effector duality can be kinetically and mechanistically uncoupled. Whether this also involves two distinct complexes is an important question to be addressed in future work. Moreover, our *atg5* KO data support feedback of autophagy on pTBK1. A negative feedback loop like this would help to keep inflammation at bay [67]. It was recently reported that active TBK1 is specifically degraded by autophagy in a process involving the ubiquitin ligase NEDD4.1. Since we have previously shown that NEDD4 family ligases are specifically recruited to the site of Ad-induced damaged membrane [14,35] it would be interesting to study if Ad controls TBK1 stability to modulate the autophagic response. An important observation is that Ads do not inhibit autophagy activation but interfere with its progression until they reach the safety of the cytosol. Activating autophagy may well be beneficial for the virus in subsequent steps either by limiting the interferon response or by providing additional transport means [14,68]. A proviral role for autophagy in Ad entry was recently shown for C-type Ad infections where starvation induced autophagy enhanced E1A expression, replication and formation of progeny virus [69,70].

Our data suggest that the cellular response to viral and bacterial membrane damage follows common principles probably extending to sterile membrane damage [15]. We tested this common role by comparing endosomal damage by Ad with lysosomal damage by LLOMe. In both cases, membrane damage results in galectin recruitment, although the extent and overlap of Gal3 and Gal8 recruitment were different for endosomal and lysosomal damage, perhaps reflecting differences in the glycoprotein composition of the damaged membranes. In contrast, TBK1 was recruited to both ruptured endosomes and ruptured lysosomes albeit less to lysosomes than endosomes. The similarity of the response, despite the different membrane damage trigger used further underlines the common role of TBK1 in membrane damage recognition. Highly localized recruitment to membrane damage, whatever its origin, coupled with TBK1 activation, provide the cell with a universal, physically constrained, damage-adapted mechanism to eliminate membrane remnants and associated pathogens by autophagy. It is conceivable that negative feedback of TBK1 on itself is held in check by the same autophagy response to allow rapid termination of the signal when the damaged membrane has been cleared. Taken together, our results provide the first detailed analysis of the cellular response to viral membrane damage. We find that TBK1 is an essential factor in the sensing of Ad-induced membrane damage and orchestrates a cell-adapted autophagy response. The similarity of the anti-viral response to the response to other membrane insults such as bacterial invasion and sterile membrane damage makes us postulate that cells possess a broadly acting, evolutionarily conserved, membrane damage response.

## Materiel and methods

### Cell culture

U2OS cells (ATCC HTB-96, kindly provided by M. Piechaczyk, IGMM, Montpellier, France) and HEK293-αvβ5 cells (ATCC CRL-1573, kindly provided by G. Nemerow, Scripps Research Institute, La Jolla, USA) were grown in Dulbecco’s modified Eagle medium (DMEM) GlutaMAX (Gibco, 31966–021) supplemented with 10% of fetal calf serum (Eurobio), 100 U/mL of penicillin and 100 μg/mL of streptomycin (Gibco, 15140122) in a 5% CO_2_ atmosphere at 37°C. All CRISPR/Cas9 KO U2OS cells were grown in the same conditions, in presence of 2 μg/mL of puromycin (Invivogen, ant-pr-1) and 20 μg/mL blasticidin (Invivogen, ant-bl-1).

### Assays involving transient transfection

Transient transfection assays were performed in 6-well plate (2.5 x 10^5^ cells/well) using Lipofectamine 2000 (Invitrogen, 11668–027) in Opti-MEM medium (Gibco, 51985–026) according to the manufacturer’s instructions using 1 μg of plasmid. Three hours post-transfection, Opti-MEM was replaced by complete DMEM and cells were kept overnight at 37°C and used for downstream infection assays.

For siRNA mediated depletion, U2OS cells were seeded in 6-well plate (2.5 x 10^5^ cells/well) and two consecutive rounds of siRNA transfection were done 24 h and 48 h post seeding. One hundred pmol of each siRNA were transfected using Lipofectamine RNAi max (Invitrogen, 13778–030) in Opti-MEM according to manufacturer’s instructions. Three hours post-transfection, Opti-MEM was replaced by complete DMEM and cells were transfected again the next day. The poly(I:C) treatment was performed 3 h by transfecting 2 μg/mL of poly(I:C) (Invivogen, tlrl-pic) using the Lipofectamine RNAi max protocol.

### Plasmids and siRNA

siRNA sequences used in this study are list in **Table 1**.

**Table 1:** List of siRNA sequences used.

Target	Sequences	Reference
Control	5’-AGG UAG UGU AAU CGC CUU G-3’	[14]
Galectin 3	5′-AAG CCC AAU GCA AAC AGA AUU GCU U-3’ 5′-GAG AAC AAC AGG AGA GUC AUU GUU U-3’	[14]
Galectin 8	5′-CCC ACG CCU GAA UAU UAA AGC AUU U-3’ 5′-GGA CAA AUU CCA GGU GGC UGU AAA U-3’	[14]
Tax1BP1	5’-CAG UCU UUG GCU UAU CAA U-3’	[71]
p62	5’-GCA UUG AAG UUG AUA UCG A-3’	[14]

Human WT TBK1 plasmids were constructed by Gateway recombination from a human orfeome library to generate pcDNA5-TBK1-GFP-3xFlag (provided by the Montpellier genomic collections platform, https://www.igmm.cnrs.fr/service/collection-genomique-de-montpellier-mgc/). TBK1 mutant expressing plasmids were constructed from pcDNA5-TBK1-GFP-3xFlag by site-directed mutagenesis using primers described in **Table 2**. The presence of the mutations was verified by sequencing.

**Table 2:** List of primers used for TBK1 mutagenesis.

Mutant	Forward primer	Reverse primer
S172A	5’-TGATGATCAGTTTGTTGCTCTGTATGGCACAGAAG-3’	5’-CTTCTGTGCCATACAGAGCAACAAACTGATCATCA-3’
K38M	5’-GGTGATTTATTCGCGATCATGGTATTTAATAAC-3’	5’-GTTATTAAATACCATGATCGCGAATAAATCACC-3’

### Virus production

All Ad vectors used in this study were replication deficient derivatives of human HAd-C5 deleted for the E1 and E3 regions, substituted in the E1 region with either an EGFP or a mCherry expression cassette (BxAd5-WT-GFP/mCherry). BxAd5-M1-GFP with a mutation in the protein VI gene [35] and BxAd5-*ts1*-GFP with a mutation in the viral protease gene [11] were also used. In the manuscript, we refer to these vectors as “WT”, “M1” and “TS1”. All vectors were produced in HEK293-α_v_β_5_ cells and purified using double CsCl_2_ banding [72]. Virus growth was performed at 37°C, except for TS1 which was grown at the non-permissive temperature of 38.5°C and the TS1-phenotype was verified by Coomassie gel analysis for unprocessed virion proteins [73]. Purified virus particles were quantified using the OD260 method (1 OD_260_ = 1.16 x 10^12^ physical particles[pp]/mL) [74] and where applicable, concentrated purified vector preparations were labeled using the Alexa Fluor Microscale labeling kit (Life Technologies, A30006), as described [75].

### Viral infections

Infections were performed for 30 min at 37°C using an inoculum of 1.8 x 10^9^ pp/mL for western blot and 6 x 10^8^ pp/100 μL for immunofluorescence, corresponding to ~5000 physical particles per cell (pp/cell). After 30 min the inoculum was removed and replaced with pre-warmed complete DMEM. Cells were incubated at 37°C for time course analysis (counting from virus addition) before cells were being further processed for western blot or immunofluorescence analysis. This high dose/short infection protocol at 37°C provided highly reproducible semi-synchronous infections suitable for time course analysis.

### Western blot

Cells were grown in 6-well plate (2.5 x 10^5^ cells/well) and infected the following day with 5000 pp/cell using the synchronous infection protocol as described above. At a given time point, cells were washed with PBS (Gibco, 70013–016) and harvested using PBS with 0.6 mM EDTA followed by a centrifugation for 5 min at 5000 g at 4°C. Cell pellets were lysed in 160 μL of lysis buffer (25 mM Tris pH7.4, 150 mM NaCl; 1 mM EDTA pH8; 5% glycerol; 1% NP40; 1 mM PMSF) supplemented with phosphatase inhibitors 1 and 2 (Sigma, P2850 and P5726). Clarified lysates were denatured in loading buffer (50 mM Tris pH 6.8, 2% SDS, 10% glycerol, 1% β-mercaptoethanol, 0.05% bromophenol blue) and separated by denaturing polyacrylamide gel electrophoresis (SDS-PAGE) and transferred on 0.2 μm nitrocellulose membrane (GE Healthcare, 10600001). Membranes were blocked for 30 min in saturation buffer (5% BSA, 0.1% Tween20 in TBS) followed by overnight incubation with primary antibodies (see **Table 3**) at 4°C and 1 h incubation with HRP conjugated secondary antibodies (Sigma) at room temperature. Immune-complexes were revealed by chemiluminescence (ECL Femto, Thermoscientific) using either a LAS 4010 camera (GE Healthcare) or Chemidoc MP Imaging System (Biorad) and analyzed with Image J software. Band intensity was measured by densitometry and the signal was normalized to actin.

### Immunofluorescence

Cells were grown on glass coverslips in 12-well plate (0.8 x 10^5^ cells/well) and infected the next day using 5000 pp/cell in a synchronous infection as described above. At a given time point, coverslips were washed with PBS and fixed with 4% PFA (PFA-EM Grade, Delta microscopy) for 20 min. Cells were permeabilized and blocked for 15 min using a single IF buffer (10% FCS, 0.05% Saponin in PBS). Primary antibodies and AlexaFluor conjugated secondary antibodies (see **Table 3**) were diluted in IF buffer and consecutively applied to cells for 1 h at 37°C in humidity chamber. Cells were mounted using fluorescent mounting medium (Dako, S3023) in presence of 1 μg/mL of DAPI (Sigma), and analyzed by confocal microscopy.

### Confocal microscopy and image analysis

Confocal images were acquired using a SP5 or SP8 confocal microscope (Leica), available through the Bordeaux Imaging Center (BIC) at Bordeaux University. Focal sections were acquired every 0.3 μm at 16 bits and 72 nm pixel size resolution using a 63x oil immersion objective. Images from at least 30 cells per condition and time point were analyzed using Image J software. Signals of interest were quantified using a semi-automatic macro (available upon request). Briefly, Z-projections of different focal planes were performed and regions of interest (ROI) were manually inserted for individual cell detection. ROI object quantifications for each channel were done automatically with appropriate predefined threshold and sizing.

### Antibodies

Primary antibodies used for western blot and immunofluorescences analysis are listed in **Table 3**.

**Table 3:** List of antibodies used and their dilution.

Target	Reference	Dilution for WB	Dilution for IF
pTBK1 (S172)	Cell Signaling Technology, 5483	1/1000	1/50
TBK1	Cell Signaling Technology, 3504	1/1000	
Actin	Merck, ab1501	1/2000	
mRFP	Chromotek, clone 5F8	1/1000	
Galectin8	R&D system, AF1305		1/200
Galectin8	Abcam, ab42879	1/500	
NDP52	Abcam, ab68588	1/1000	1/200
Tax1BP1	Cell Signaling Technology, 5105	1/1000	
p62	BD transduction, 610832	1/500	1/250
VI	Merck, MABF2196 [11]		1/200
IRF3	Cell Signaling Technology, 11904		1/400

### Flow cytometry

Cells were seeded in 24-well plate (1 x 10^5^ cells/well) and pre-treated (when required) for 3 h with MRT67-307 inhibitor (Sigma, SML0702) used at a concentration from 5 μM to 25 μM. Transduction was performed using 50 pp/cell of WT or M1 vectors expressing either GFP or mCherry, in the presence or absence of inhibitor. Three hours post infection, complete DMEM containing virus was removed and replaced by complete DMEM overnight at 37°C. Twenty-four hours later, cells were washed in PBS and collected using 200 μL of trypsin and 300 μL of PBS with 0.6 mM EDTA and analyzed by flow cytometry for GFP and/or mCherry expression using FACS Canto I cytometer (BD Biosciences). For each condition, 10000 cells were analyzed using FACS DIVA software.

### Statistical analysis

Graph design and statistical analysis were performed with GraphPad Prism (version 7). Data are shown as mean and +/-SD. For immunofluorescence analysis, n > 30 analyzed cells. P values were calculated using either Student t-test or one way ANOVA, followed by Tukey’s or Dunnett’s post-test (n.s = not significant, * = P<0.05; ** = P<0.01; *** = P<0.001; **** = P<0.0001).

## Supporting information

S1 Data The supporting information file contains the raw data and statistical analysis of Figs 1B, 1C, 1D, 1E, 2B, 2C, 2D, 2E, 2F, 2G, 3C, 3D, 3G, 4B, 4C, 4E, 4F, 4H, 4I, 5A, 5B, 5E, 5G, 5H, 6B, 6F, 6G, 7C, 7D, 7F, 7G, 8B, 8D, 8E, 8G, 8H, 9C, 9D, 9E, 9F. (XLSX)
